# Machine learning prediction models for mechanical ventilation requirement in patients with embolic stroke: a retrospective cohort study based on MIMIC-IV

**DOI:** 10.3389/fmed.2026.1945368

**Published:** 2026-09-09

**Authors:** Li Ma, Xuhui Liu, Ruiling Nan, Hui Yan, Xinglei Wang, Haixia Chen, Xinman Dou, Xujie Wang

**Affiliations:** 1 Department of Oncology Surgery, Lanzhou University Second Hospital, Lanzhou, Gansu, China; 2 Department of Neurology, Lanzhou University Second Hospital, Lanzhou, Gansu, China; 3 Department of Nursing, Lanzhou University Second Hospital, Lanzhou, Gansu, China; 4 Department of Cardiology, Lanzhou University Second Hospital, Lanzhou, Gansu, China; 5 School of Nursing, Lanzhou University, Lanzhou, Gansu, China; 6 Department of Emergency ICU, The Affiliated Hospital of Qinghai University, Xining, China

**Keywords:** embolic stroke, machine learning, mechanical ventilation, MIMIC-IV, prediction model

## Abstract

**Objective:**

This study seeks to develop and compare four machine learning models using the MIMIC-IV database to identify predictive variables associated with invasive mechanical ventilation (MV) requirement in patients with embolic cerebral infarction.

**Methods:**

This study included 568 patients with embolic cerebral infarction from the MIMIC-IV database. Data collected encompassed demographic information, vital signs, laboratory results, and other relevant variables. The dataset was randomly split into a development set and a validation set in a 7:3 ratio. Feature selection was performed using univariable and multivariable analyses. Four prediction models, namely decision tree (DT), logistic regression (LR), eXtreme Gradient Boosting (XGB), and Light Gradient Boosting Machine (LGBM), were developed. Model performance was assessed using receiver operating characteristic (ROC) curve analysis. Calibration was evaluated through calibration curves and Brier scores. Clinical utility was examined using decision curve analysis (DCA), and Shapley additive explanations (SHAP) were used to interpret model predictions. ICU admission was t_0_ and predictors were summarized over the first 24 h. Because exact MV-initiation times were unavailable, robustness analyses addressed optimism, class imbalance, and temporal ambiguity using repeated nested cross-validation, an all-MV benchmark, inverse-frequency weighting, and exclusion of pneumonia and log input amount.

**Results:**

Among 568 patients, 438 received invasive MV. XGB achieved an AUROC of 0.839 (95% CI, 0.796–0.883) in the development set and 0.720 (95% CI, 0.637–0.801) in the validation set; repeated nested cross-validation yielded 0.713 (95% CI, 0.665–0.757). The all-MV benchmark had a specificity of 0 and 0.500 balanced accuracy, whereas XGB achieved 0.700 and 0.659, respectively. Excluding pneumonia and log input amount reduced the nested-CV AUROC to 0.654 (95% CI, 0.595–0.703). SHAP analysis identified pneumonia, hematocrit, INR, weight, heart failure, log input amount, and glucose as the highest-importance features.

**Conclusion:**

This study developed four machine learning models to assess the necessity of mechanical ventilation support in patients with embolic cerebral infarction and compared their performance. XGB showed the most favorable overall validation profile but requires time-stamped external validation before clinical use.

## 1 Introduction

Ischemic stroke constitutes approximately 60% to 70% of all stroke cases globally and represents a predominant neurological condition responsible for mortality and disability worldwide (1). Embolic stroke, also known as embolic cerebral infarction, is the most common subtype of ischemic stroke. It is caused by emboli originating from the heart or other sources blocking the cerebral arteries. This condition is characterized by a sudden onset, severe neurological deficits and generally poor outcomes (2, 3). Patients admitted to the ICU with embolic cerebral infarction often require mechanical ventilation (MV) support to maintain vital signs. MV serves as a double-edged sword. While it offers essential life support for gas exchange in critically ill patients, it also poses risks including ventilator-associated pneumonia, delirium, and extended stays in the ICU (4–6). Thus, promptly and accurately determining if patients with embolic cerebral infarction need MV support is crucial for delivering precise medical care, efficiently allocating resources, and enhancing patient outcomes.

At present, clinical scoring systems like SOFA and APACHE II are widely used to predict the severity of illnesses; however, these tools provide general assessments of severity and are not tailored to specific diseases (7, 8). Furthermore, it is important to highlight that these scoring systems depend on linear models to determine relevant predictors, which limits their ability to detect nonlinear relationships and complex interactions between predictors (9). In recent years, machine learning algorithms have successfully identified complex nonlinear patterns and shown strong predictive capabilities, making them a promising tool for early disease detection. However, their black-box nature restricts their use in clinical settings (10). The Shapley Additive Explanation (SHAP) method provides a balanced and efficient way to break down prediction outcomes into additive parts that reflect both local and global interpretability, successfully addressing the difficulties caused by the lack of model transparency (11). This study aims to create a predictive model to determine whether patients with embolic cerebral infarction admitted to the ICU will need MV support, using the comprehensive MIMIC-IV database. Initially, data preprocessing was performed to address missing and abnormal values, followed by the selection of key predictive features through univariable and multivariable logistic regression analyses. These important factors were then used to develop predictive models employing different machine learning algorithms, including decision tree (DT), logistic regression (LR), eXtreme Gradient Boosting (XGB), and Light Gradient Boosting Machine (LGBM). Furthermore, the study utilized the SHAP method to interpret and analyze the contribution of each feature.

## 2 Methods

### 2.1 Data source and study cohort

This retrospective study utilizes the MIMIC-IV (version 3.1) database (12), which was collaboratively created by the Massachusetts Institute of Technology (MIT), Beth Israel Deaconess Medical Center (BIDMC), and Philips. This database serves as a comprehensive source of critical care data. The study period spans from 2008 to 2019 and includes records of 65,366 ICU patients across 28 tables, containing detailed information on demographics, vital signs, medical histories, laboratory results, and other relevant data. Ethical approval from MIT and BIDMC ensures compliance and protects patient privacy through data anonymization, eliminating the need for explicit consent (13). Access to the database was granted for this study (Record ID: 13931651).

### 2.2 Participants

**Figure 1:**
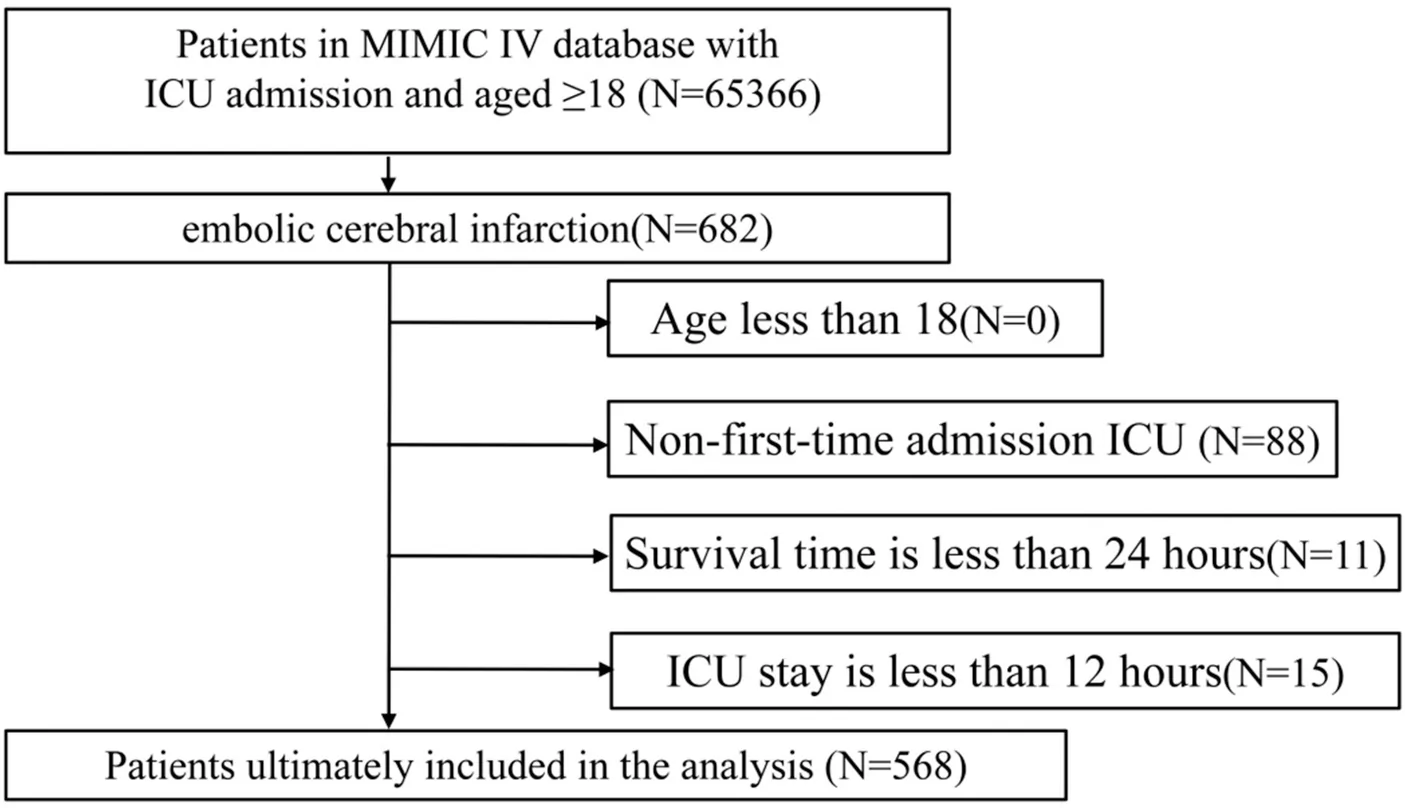
Flow chart of **participant selection**.

The study included patients aged ≥18 years with embolic cerebral infarction recorded in the MIMIC-IV database. Patients were excluded if the ICU stay was <12 h or survival after ICU admission was <24 h. For those with multiple ICU admissions, only ICU admission records from the patient's initial admission were considered. The patient screening process is shown in Figure 1.

### 2.3 Data extraction and outcomes

Patients with embolic cerebral infarction were identified using ICD-9-CM code 434.11 (cerebral embolism with cerebral infarction), as documented in the supplied extraction dictionary. No ICD-10-CM code was documented or used for the analytic cohort. ICU admission was defined as t_0_, and predictor variables were summarized during [t_0_, t_0_ _+_ _24 h_) using Structured Query Language (SQL) in PostgreSQL. The extracted variables include: (a) Demographic and clinical information such as age, gender, and weight; (b) Chronic conditions and diagnoses recorded by the end of the first 24 h, including hypertension, diabetes, heart failure (HF), myocardial infarction (MI), pneumonia, chronic obstructive pulmonary disease (COPD), and hyperlipidemia; (c) Vital signs recorded in the ICU during the first 24 h, including average body temperature (T), mean arterial pressure (MAP), systolic blood pressure (SBP), diastolic blood pressure (DBP), SpO2, heart rate (HR), respiratory rate (RR), fluid input and output volumes; (d) Laboratory test results within the first 24 h in the ICU, such as hemoglobin (Hb), platelet count (PLT), red blood cell distribution width (RDW), white blood cell count (WBC), red blood cell count (RBC), hematocrit, international normalized ratio (INR), prothrombin time (PT), activated partial thromboplastin time (APTT), blood urea nitrogen (BUN), serum creatinine concentration (Cr), anion gap (AG), calcium (Ca), chloride (Cl), glucose concentration (Glu), sodium (Na) and potassium (K); (e) Medication use: aspirin, clopidogrel, and statins.

To reduce the impact of missing data on model construction, random-forest imputation was used for variables with <30% missingness, whereas variables with >30% missingness were excluded. The missing rate of included variables is shown in Supplementary Figure S1. For skewed features, we applied specific transformation techniques: a cubic transformation was used when skewness was ≥1, and a logarithmic transformation was used when skewness was <1. To address multicollinearity, we employed multivariable stepwise logistic regression.

The primary outcome was a binary indicator of invasive mechanical ventilation during the ICU stay. The supplied final analytic file did not retain exact MV-initiation timestamps; therefore, strict temporal ordering between first-24-h predictors and the outcome could not be verified. This limitation was examined in a temporal-sensitivity analysis that excluded pneumonia and log input amount(Supplementary Figure S2). MV was defined solely as invasive mechanical ventilation, including positive pressure ventilation delivered via endotracheal intubation or tracheostomy. Brief ventilation provided for airway protection or procedural sedation was also included; however, non-invasive mechanical ventilation alone (such as continuous positive airway pressure or bilevel positive airway pressure) was not included in the outcome.

### 2.4 Model development and tuning

Prior to constructing the model, univariable and multivariable logistic regression analyses were conducted to select candidate predictors. Multicollinearity was evaluated after definition of the candidate predictor set but before final-model estimation using Spearman correlations, VIF, and tolerance values. This study employed four machine learning algorithms to develop predictive models, including LR, DT, LGBM, and XGB (14, 15). We used grid search to tune hyperparameters to enhance the model's performance. After training the model on the development dataset, we conducted internal validation using k-fold cross-validation (16). This approach effectively assesses model performance by training and testing it on different portions of the training data, which provides an internal performance estimate but does not eliminate overfitting. The evaluation metrics included the area under the receiver operating characteristic curve (AUROC), confusion matrix, accuracy, recall, specificity, precision, and F1 score. For the AUROC, a 95% confidence interval(CI) was calculated using the bootstrap resampling technique. Additionally, calibration curves, Brier scores, and decision curve analysis (DCA) were used together to evaluate the model. The calibration curve visually examines how well predicted probabilities align with actual outcomes, while the Brier score provides a numerical measure of the accuracy of these probability predictions. DCA assesses the clinical usefulness of the model by considering the balance of benefits and risks in decision-making. In addition, to assess whether transient ventilatory support introduced heterogeneity into the outcome definition, we performed sensitivity analyses excluding mechanically ventilated patients with ventilation durations <12 h and <24 h, while retaining all non-ventilated patients and the original development–validation allocation. The same seven-predictor XGB model was refitted and evaluated for discrimination, calibration, and clinical utility.

All primary hyperparameter tuning was confined to the development cohort, and the held-out validation cohort was not used for model selection. To examine XGB fitting and tuning optimism, we performed stratified nested fivefold cross-validation with 10 repeats. Within each outer training fold, inner fivefold cross-validation selected the number of boosting rounds from 5, 10, 15, 20, 25, 30, 40, 50, 75, and 100; only outer test-fold predictions contributed to performance estimates. Because only the final imputed, transformed predictor matrix was available, imputation, transformation, and predictor selection were not re-estimated within folds; thus, this analysis evaluated model fitting and tuning conditional on fixed preprocessing. Given the 77.1% MV prevalence, we emphasized AUROC, AUPRC, balanced accuracy, sensitivity, specificity, and Brier score, compared XGB with an all-MV benchmark, and evaluated inverse-frequency weighting. Temporal sensitivity was assessed by excluding pneumonia and log input amount.

### 2.5 Model interpretation and feature importance

We utilized SHAP values to interpret the predictions of the selected XGB model (17). These values quantify each feature's contribution to the model output. Using the “shapviz” package in R, we calculated and visualized the SHAP values, thereby enhancing the interpretability of individual predictions.

### 2.6 Statistical analysis

All statistical analyses and data visualizations were performed using R version 4.3.1. Quantitative data are presented as mean ± standard deviation (SD), skewed data as median (interquartile range [IQR]), and categorical variables as frequency and percentage. Comparisons between groups were conducted using t-tests, Wilcoxon rank-sum tests, chi-square tests, or Fisher's exact tests, as appropriate. Multicollinearity among candidate predictors was assessed using the variance inflation factor (VIF). A VIF value exceeding 10 was considered indicative of significant multicollinearity, and variables exceeding this threshold were excluded from the final multivariable model. *Post-hoc* robustness analyses were performed in Python 3.13 using scikit-learn 1.8.0, XGB 3.1.3, and statsmodels 0.14.6. Stratified patient-level bootstrap resampling provided 95% CI, and paired bootstrap resampling was used for AUROC differences.

This study used several R packages for data processing and visualization, such as ‘tidyverse’ for managing data, ‘pROC’ for analyzing ROC curves, ‘rms’ for regression modeling, and ‘rmda’ for conducting decision curve analysis. *P* < 0.05 was regarded as statistically significant. All statistical tests were two-sided.

## 3 Results

### 3.1 Participant characteristics

This study categorized 568 eligible participants into a development group (*n* = 397) and a validation group (*n* = 171). Out of these 568 participants, 438 required MV support, with 307 in the development group and 131 in the validation group. Table 1 compares MV and non-MV patients. Supplementary Table S1 compares the development and validation groups; most variables were similar, although K and pneumonia differed (*P* = 0.010 and *P* = 0.013, respectively).

**Table 1:** Characteristics of MV and non-MV patients of the MIMIC-IV database.

Variables	Total (*n* = 568)	Non-MV (*n* = 130)	MV (*n* = 438)	Statistic	*P*
Age, M (Q₁, Q₃)	71.50 (60.00, 83.00)	69.00 (56.00, 81.00)	73.00 (61.00, 83.00)	Z = −1.68	0.092
Gender, *n* (%)				*χ*^2^ = 0.17	0.677
Female	280 (49.30)	62 (47.69)	218 (49.77)		
Male	288 (50.70)	68 (52.31)	220 (50.23)		
Weight, M (Q₁, Q₃)	77.35 (64.55, 93.03)	71.25 (59.92, 86.40)	78.70 (66.71, 95.94)	Z = −3.69	<.001
Hypertension, *n* (%)				χ^2^ = 0.43	0.511
No	237 (41.73)	51 (39.23)	186 (42.47)		
Yes	331 (58.27)	79 (60.77)	252 (57.53)		
Diabetes, *n* (%)				χ^2^ = 1.39	0.238
No	414 (72.89)	100 (76.92)	314 (71.69)		
Yes	154 (27.11)	30 (23.08)	124 (28.31)		
HF, *n* (%)				χ^2^ = 10.27	0.001
No	424 (74.65)	111 (85.38)	313 (71.46)		
Yes	144 (25.35)	19 (14.62)	125 (28.54)		
MI, *n* (%)				χ^2^ = 2.23	0.135
No	550 (96.83)	129 (99.23)	421 (96.12)		
Yes	18 (3.17)	1 (0.77)	17 (3.88)		
Pneumonia, *n* (%)				χ^2^ = 29.96	<.001
No	397 (69.89)	116 (89.23)	281 (64.16)		
Yes	171 (30.11)	14 (10.77)	157 (35.84)		
Hyperlipidemia, *n* (%)				χ^2^ = 7.19	0.007
No	331 (58.27)	89 (68.46)	242 (55.25)		
Yes	237 (41.73)	41 (31.54)	196 (44.75)		
COPD, *n* (%)				χ^2^ = 0.01	0.926
No	561 (98.77)	129 (99.23)	432 (98.63)		
Yes	7 (1.23)	1 (0.77)	6 (1.37)		
T, M (Q₁, Q₃)	36.78 (36.44, 37.11)	36.78 (36.50, 37.06)	36.78 (36.39, 37.17)	Z = −0.54	0.592
MAP, M (Q₁, Q₃)	87.00 (74.00, 100.00)	87.50 (75.00, 97.00)	87.00 (74.00, 102.00)	Z = −0.10	0.918
SBP, Mean ± SD	135.88 ± 26.89	135.45 ± 24.40	136.01 ± 27.62	t = −0.21	0.836
DBP, M (Q₁, Q₃)	71.00 (59.00, 86.00)	71.50 (60.00, 86.00)	71.00 (58.00, 86.00)	Z = −0.33	0.743
SpO2, M (Q₁, Q₃)	98.00 (96.00, 100.00)	98.00 (96.00, 99.00)	98.00 (95.00, 100.00)	Z = −0.12	0.902
HR, M (Q₁, Q₃)	84.00 (72.00, 100.00)	81.00 (71.00, 92.75)	85.00 (73.00, 102.00)	Z = −2.10	0.036
RR, M (Q₁, Q₃)	18.00 (15.00, 22.00)	18.00 (15.00, 21.00)	18.00 (15.00, 22.00)	Z = −0.63	0.531
Log Input Amount, M (Q₁, Q₃)	7.95 (7.55, 8.39)	7.73 (7.36, 8.19)	8.00 (7.60, 8.44)	Z = −3.70	<.001
Log Output Amount, M (Q₁, Q₃)	7.59 (7.17, 7.99)	7.55 (7.08, 7.97)	7.59 (7.20, 8.00)	Z = −1.04	0.298
Hb, Mean ± SD	11.68 ± 2.32	12.16 ± 2.12	11.54 ± 2.36	t = 2.70	0.007
PLT, M (Q₁, Q₃)	211.00 (158.00, 275.25)	219.00 (179.25, 285.50)	208.00 (153.00, 273.75)	Z = −2.19	0.028
RDW, M (Q₁, Q₃)	14.10 (13.40, 15.00)	13.90 (13.12, 14.57)	14.10 (13.40, 15.17)	Z = −2.28	0.023
WBC, M (Q₁, Q₃)	10.50 (8.10, 13.50)	9.95 (7.78, 12.47)	10.70 (8.20, 13.70)	Z = −2.31	0.021
RBC, M (Q₁, Q₃)	3.94 (3.36, 4.39)	4.04 (3.69, 4.47)	3.89 (3.32, 4.35)	Z = −2.60	0.009
Hematocrit, Mean ± SD	34.94 ± 6.67	36.29 ± 5.87	34.54 ± 6.84	t = 2.87	0.004
INR, M (Q₁, Q₃)	1.20 (1.10, 1.40)	1.20 (1.10, 1.20)	1.20 (1.10, 1.40)	Z = −4.42	<.001
PT, M (Q₁, Q₃)	13.70 (12.47, 15.40)	13.10 (11.90, 13.97)	13.80 (12.70, 15.70)	Z = −4.81	<.001
APTT, M (Q₁, Q₃)	29.60 (26.48, 34.92)	29.60 (26.72, 32.32)	29.60 (26.42, 35.60)	Z = −1.77	0.077
BUN, M (Q₁, Q₃)	18.00 (13.00, 27.00)	17.50 (12.00, 25.00)	18.00 (13.00, 28.00)	Z = −2.08	0.037
Cr, M (Q₁, Q₃)	0.90 (0.70, 1.30)	0.90 (0.70, 1.20)	0.90 (0.80, 1.30)	Z = −1.49	0.137
AG, M (Q₁, Q₃)	14.00 (12.00, 16.00)	14.00 (13.00, 16.00)	14.00 (12.00, 16.00)	Z = −0.86	0.388
Ca, M (Q₁, Q₃)	8.50 (8.00, 8.90)	8.80 (8.33, 9.10)	8.50 (8.00, 8.88)	Z = −4.42	<.001
Cl, M (Q₁, Q₃)	105.00 (102.00, 109.00)	105.00 (102.00, 108.00)	106.00 (102.00, 109.75)	Z = −1.38	0.167
Glu, M (Q₁, Q₃)	122.00 (105.00, 152.00)	118.00 (100.00, 139.00)	124.00 (106.00, 160.00)	Z = −2.60	0.009
Na, M (Q₁, Q₃)	139.00 (137.00, 141.25)	139.00 (136.00, 141.00)	139.00 (137.00, 142.00)	Z = −1.53	0.127
K, M (Q₁, Q₃)	4.00 (3.70, 4.40)	3.90 (3.62, 4.30)	4.00 (3.70, 4.40)	Z = −1.22	0.223
Aspirin, *n* (%)				χ^2^ = 1.57	0.210
No	134 (23.59)	36 (27.69)	98 (22.37)		
Yes	434 (76.41)	94 (72.31)	340 (77.63)		
Clopidogrel, *n* (%)				χ^2^ = 1.56	0.212
No	559 (98.42)	130 (100.00)	429 (97.95)		
Yes	9 (1.58)	0 (0.00)	9 (2.05)		
Statin, *n* (%)				χ^2^ = 0.19	0.662
No	460 (80.99)	107 (82.31)	353 (80.59)		
Yes	108 (19.01)	23 (17.69)	85 (19.41)		

t: t-test, Z: Mann–Whitney test, χ^2^: Chi-square test.

SD, standard deviation; M, median; Q₁, first quartile; Q₃, third quartile.

### 3.2 Determination of the candidate prediction set

Univariable logistic regression was performed. The findings revealed that HF, pneumonia, RBC, Hb, hematocrit, Ca, Glu, PT, INR, weight, and log input amount were significantly associated with the requirement for MV (*P* < 0.05). A multivariable logistic regression model was then fitted. HF, pneumonia, PT, log input amount, weight, hematocrit, INR, and Glu were retained as candidate predictors. These results are presented in Table 2.

**Table 2:** Univariable and multivariable logistic regression analyses of predictors for MV.

Variables	Univariable					Multivariable				
	*β*	SE	Z	*P*	OR (95% CI)	β	SE	Z	*P*	OR (95% CI)
Aspirin										
No					1.00 (Reference)					
Yes	0.23	0.28	0.81	0.418	1.25 (0.73∼2.16)					
Clopidogrel										
No					1.00 (Reference)					
Yes	14.36	594.16	0.02	0.981	17,21,605.17 (0.00∼Inf)					
Statin										
No					1.00 (Reference)					
Yes	0.24	0.32	0.74	0.457	1.27 (0.68∼2.35)					
Hypertension										
No					1.00 (Reference)					
Yes	−0.08	0.24	−0.32	0.750	0.93 (0.57∼1.49)					
Gender										
Female					1.00 (Reference)					
Male	0.06	0.24	0.27	0.790	1.07 (0.67∼1.71)					
Diabetes										
No					1.00 (Reference)					
Yes	0.20	0.28	0.70	0.483	1.22 (0.70∼2.11)					
HF										
No					1.00 (Reference)					1.00 (Reference)
Yes	0.95	0.32	2.91	0.004	2.57 (1.36∼4.86)	0.87	0.36	2.45	0.014	2.40 (1.19∼4.83)
Pneumonia										
No					1.00 (Reference)					1.00 (Reference)
Yes	1.49	0.34	4.34	<.001	4.42 (2.26∼8.66)	1.30	0.36	3.67	<.001	3.68 (1.84∼7.39)
MI										
No					1.00 (Reference)					
Yes	15.38	692.69	0.02	0.982	47,74,991.43 (0.00∼Inf)					
Hyperlipidemia										
No					1.00 (Reference)					
Yes	0.45	0.25	1.82	0.069	1.57 (0.97∼2.57)					
COPD										
No					1.00 (Reference)					
Yes	14.36	594.16	0.02	0.981	1721605.17 (0.00∼Inf)					
WBC	0.04	0.03	1.69	0.092	1.04 (0.99∼1.10)					
PLT	−0.00	0.00	−1.65	0.100	1.00 (1.00∼1.00)					
RBC	−0.33	0.16	−2.08	0.037	0.72 (0.52∼0.98)					
Hb	−0.12	0.05	−2.28	0.022	0.89 (0.80∼0.98)					
RDW	0.11	0.07	1.55	0.121	1.12 (0.97∼1.28)					
Hematocrit	−0.04	0.02	−2.33	0.020	0.96 (0.92∼0.99)	−0.04	0.02	−1.65	0.099	0.97 (0.93∼1.01)
Na	0.02	0.03	0.58	0.562	1.02 (0.96∼1.07)					
K	0.14	0.19	0.72	0.474	1.15 (0.78∼1.68)					
Ca	−0.44	0.16	−2.73	0.006	0.64 (0.47∼0.88)					
Cl	0.03	0.02	1.22	0.221	1.03 (0.98∼1.07)					
Glu	0.01	0.00	2.30	0.022	1.01 (1.01∼1.01)	0.00	0.00	1.42	0.155	1.00 (1.00∼1.01)
PT	0.20	0.06	3.43	<.001	1.22 (1.09∼1.36)	0.56	0.24	2.33	0.020	1.74 (1.09∼2.78)
APTT	0.00	0.01	0.63	0.527	1.00 (0.99∼1.02)					
INR	1.79	0.58	3.10	0.002	6.01 (1.94∼18.69)	−4.57	2.41	−1.89	0.058	0.01 (0.00∼1.18)
BUN	0.01	0.01	1.25	0.210	1.01 (0.99∼1.03)					
Cr	0.03	0.07	0.48	0.633	1.03 (0.90∼1.18)					
HR	0.01	0.01	1.86	0.063	1.01 (1.00∼1.02)					
SBP	0.00	0.00	0.83	0.404	1.00 (0.99∼1.01)					
AG	−0.00	0.04	−0.03	0.972	1.00 (0.93∼1.07)					
DBP	0.00	0.01	0.52	0.606	1.00 (0.99∼1.02)					
MAP	0.01	0.01	0.95	0.342	1.01 (0.99∼1.02)					
RR	0.02	0.02	0.89	0.376	1.02 (0.98∼1.07)					
SpO2	−0.07	0.04	−1.58	0.114	0.93 (0.86∼1.02)					
T	−0.17	0.15	−1.09	0.274	0.85 (0.63∼1.14)					
Weight	0.01	0.01	2.36	0.018	1.01 (1.01∼1.03)	0.01	0.01	2.29	0.022	1.01 (1.01∼1.03)
Age	0.01	0.01	1.87	0.061	1.01 (1.00∼1.03)					
Log Input Amount	0.40	0.15	2.62	0.009	1.49 (1.11∼2.01)	0.30	0.17	1.80	0.072	1.36 (0.97∼1.89)

### 3.3 Diagnosis of multicollinearity

In the initial candidate model, PT and INR showed severe multicollinearity, with VIF values of 21.473 and 21.599, respectively, and a Spearman correlation coefficient of 0.955. After PT was excluded, all VIF values ranged from 1.017 to 1.111, indicating no material multicollinearity in the final predictor set (Supplementary Figure S3 and Supplementary Table S2). The refitted seven-predictor model was significant overall (likelihood-ratio *χ*^2^ = 54.92, df = 7, *P* < 0.001). HF (adjusted OR, 2.301; 95% CI, 1.154–4.587), pneumonia (adjusted OR, 3.770; 95% CI, 1.883–7.549), and weight (adjusted OR, 1.014; 95% CI, 1.001–1.027) were independently associated with MV. Complete model parameters are reported in Table 3 and Supplementary Figure S4.

**Table 3:** Refitted multivariable logistic regression model after exclusion of PT.

Variables	β	SE	Adjusted OR (95% CI)	*P* value
HF	0.833	0.352	2.301 (1.154–4.587)	0.018
Pneumonia	1.327	0.354	3.770 (1.883–7.549)	<0.001
Hematocrit	−0.040	0.021	0.961 (0.922–1.002)	0.063
Glu	0.004	0.003	1.004 (0.998–1.009)	0.190
Weight	0.014	0.006	1.014 (1.001–1.027)	0.029
Log input amount	0.261	0.166	1.299 (0.937–1.799)	0.116
INR	0.926	0.567	2.523 (0.831–7.662)	0.102

### 3.4 Model development and validation

**Figure 2:**
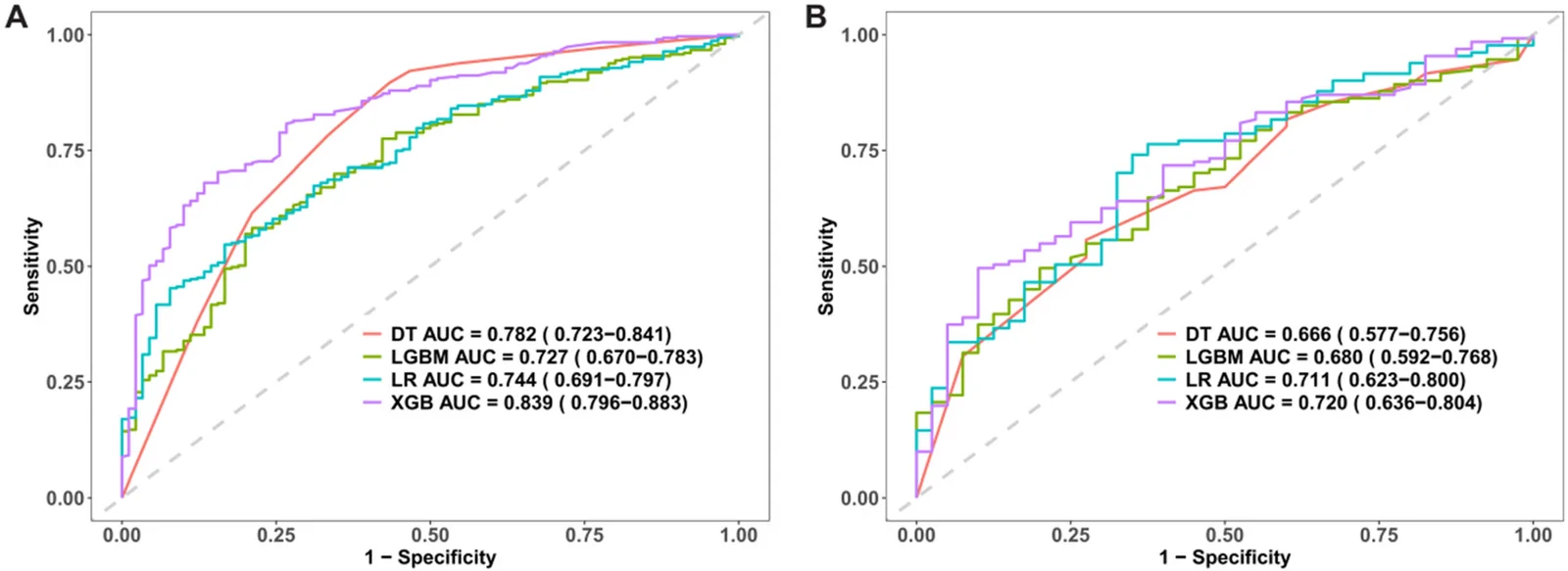
Receiver operating characteristic (ROC) curves of the four machine learning models for predicting MV requirement in patients with embolic cerebral infarction. DT, decision tree; LGBM, Light Gradient Boosting Machine; LR, logistic regression; XGB, eXtreme Gradient Boosting. **(A)** Development group; **(B)** validation group.

Based on the feature variables, we developed four machine learning models—DT, LR, XGB, and LGBM—to determine whether patients with embolic cerebral infarction admitted to the ICU require MV support. Figure 2 presents a comparison of the discriminative performance of these models using the ROC curve. Among them, XGB had the highest development-set AUROC, 0.839 (95% CI, 0.796–0.883), and was selected for subsequent interpretation. While the other models also demonstrated good predictive capabilities, their performance ranked as follows in descending order: DT (AUROC, 0.782; 95% CI, 0.723–0.841), LR (AUROC, 0.744; 95% CI, 0.691–0.797), and LGBM (AUROC, 0.727; 95% CI, 0.670–0.783). In the held-out validation set, XGB achieved an AUROC of 0.720 (95% CI, 0.637–0.801), an absolute decrease of 0.119 from the development estimate. The selected XGB hyperparameters were seed = 1234, booster = gbtree, eta = 0.3, gamma = 0.001, max_depth = 2, subsample = 0.7, colsample_bytree = 0.4, objective = binary:logistic, and validate_parameters = TRUE.

**Figure 3:**
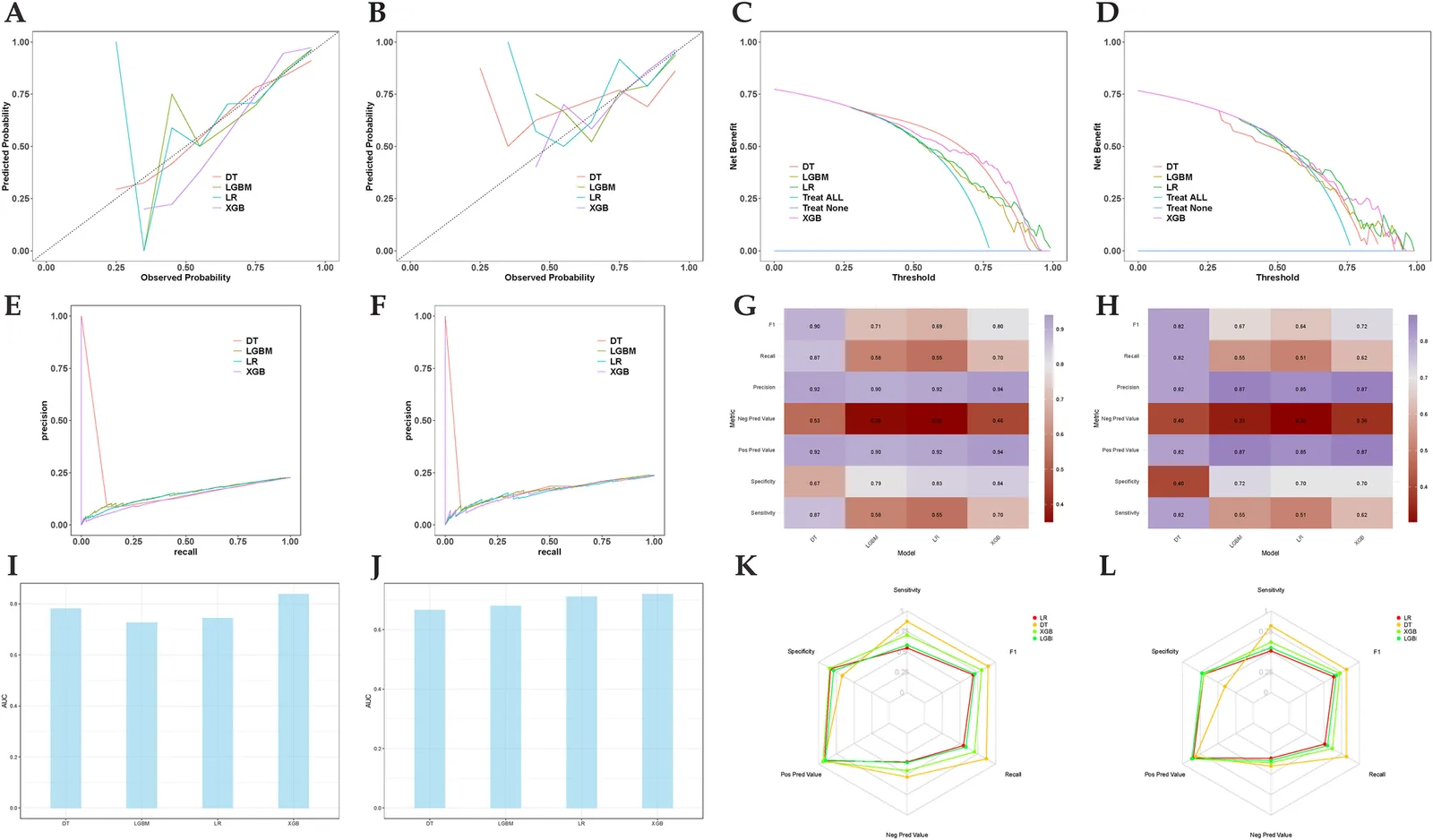
Comprehensive performance evaluation of the four machine learning models. **(A,B) Calibration curves**; **(C,D) Decision curve analysis** (DCA); **(E,F) PrecisioN–Recall** (PR) curves; **(G,H) heat maps** of confusion matrices; **(I,J) bar charts comparing the AUROC** of the four models; **(K,L) radar charts**; **Development group**: A, C, E, G, I, K; validation group: B, D, F, H, J, L.

Table 4 offers a comprehensive overview of the performance metrics for the four models. Figure 3 presents calibration, DCA, precision-recall, confusion-matrix, and AUROC comparisons. The Brier scores of the validation group for the LR, DT, LGBM, and XGB models were 0.1630, 0.1873, 0.1711, and 0.1607, respectively (Supplementary Table S3). DCA showed greater net benefit for XGB across portions of the evaluated threshold range. According to the DeLong test results on the development set, XGB AUROC was significantly higher than those of the other three models, while no significant differences were found among DT, LR, and LGBM (Supplementary Table S4). In the validation set, no statistically significant differences were observed among the models.

**Table 4:** Comparison of performance metrics across the four models.

Metric	Development set				Validation set			
Model	LR	DT	XGB	LGBM	LR	DT	XGB	LGBM
Sensitivity	0.547	0.871	0.704	0.583	0.511	0.817	0.618	0.550
Specificity	0.833	0.667	0.844	0.789	0.700	0.400	0.700	0.725
Positive predictive value	0.918	0.922	0.939	0.904	0.848	0.817	0.871	0.867
Negative predictive value	0.350	0.533	0.455	0.357	0.304	0.400	0.359	0.330
Precision	0.918	0.922	0.939	0.904	0.848	0.817	0.871	0.867
Recall	0.547	0.871	0.704	0.583	0.511	0.817	0.618	0.550
F1 score	0.686	0.896	0.804	0.709	0.638	0.817	0.723	0.673
Balanced Accuracy	0.690	0.769	0.774	0.686	0.606	0.608	0.659	0.637

Repeated nested cross-validation yielded an AUROC of 0.713 (95% CI, 0.665–0.757), an AUPRC of 0.898 (95% CI, 0.875–0.918), and a Brier score of 0.161 (95% CI, 0.153–0.169); the median selected number of boosting rounds was 20 (range, 5–100). The all-MV benchmark achieved 0.766 accuracy but a specificity of 0 and 0.500 balanced accuracy, whereas XGB achieved 0.637 accuracy, 0.700 specificity, and 0.659 balanced accuracy. Inverse-frequency weighting yielded an AUROC of 0.717 and a Brier score of 0.200. Excluding pneumonia and log input amount reduced the nested-cross-validation AUROC to 0.654 (95% CI, 0.595–0.703; difference, 0.059; bootstrap *P* < 0.001) (Supplementary Tables S5 and S6 and Supplementary Figure S5).

Subsequently, we conducted two sensitivity analyses to assess the impact of clinical heterogeneity in the MV group on the robustness of the model. Of the 438 mechanically ventilated patients in the primary cohort, 83 had a recorded ventilation duration <12 h and 160 had a duration <24 h. The corresponding restricted cohorts included 485 and 408 patients, respectively. Validation AUROCs were 0.720 (95% CI, 0.637–0.801) in the primary analysis, 0.721 (95% CI, 0.631–0.800) after excluding patients with MV durations <12 h, and 0.798 (95% CI, 0.709–0.869) after excluding patients with MV durations <24 h. AUPRC and Brier scores remained broadly comparable, calibration intercepts remained close to zero with slopes close to one, and DCA demonstrated preserved net benefit across clinically relevant threshold ranges. These findings supported the robustness of the model to stricter definitions of clinically meaningful MV (Supplementary Figures S6 and S7 and Supplementary Table S7).

### 3.5 Interpretability analysis

**Figure 4:**
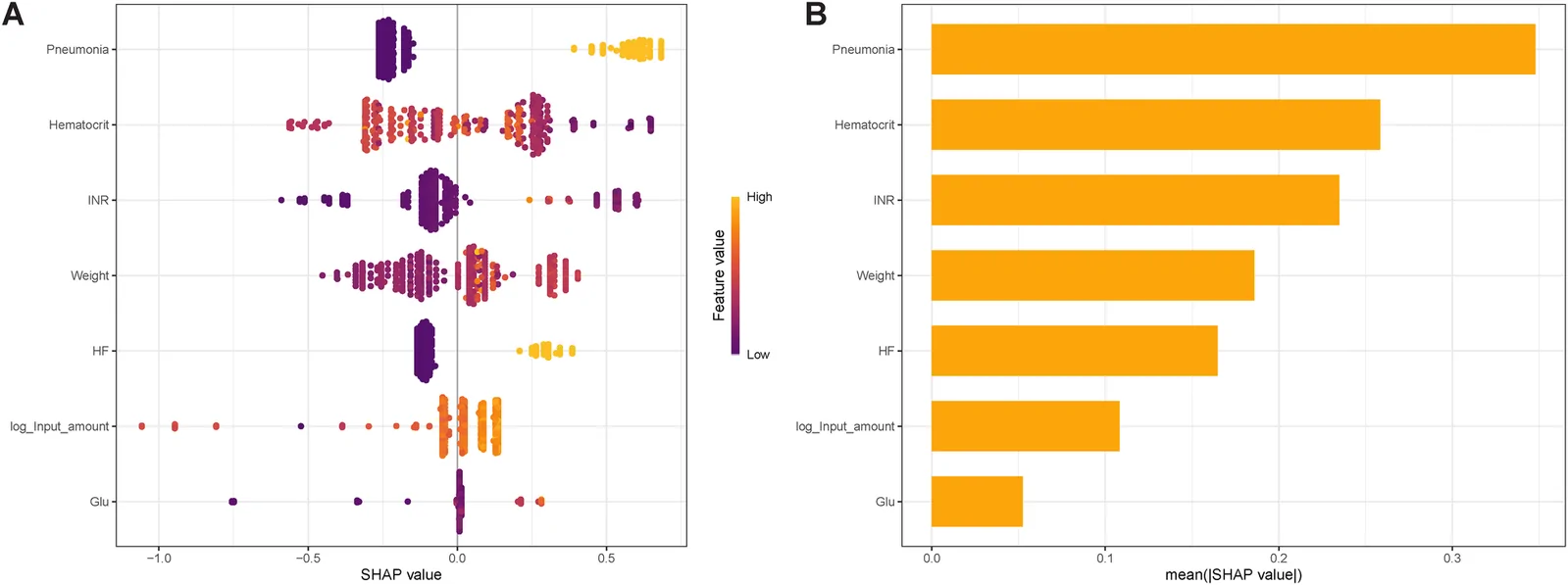
SHAP value analysis of the XGBoost model. **(A)** Summary plot of the SHAP values for the XGB model; **(B)** SHAP variable importance ranking.

SHAP analysis was used to characterize feature contributions to individual XGB predictions of MV requirement. Mean absolute SHAP importance ranked the features as follows: pneumonia, hematocrit, INR, weight, HF, total input volume, and Glu. Supplementary Figure S8 presents the feature importance rankings based on Cover, Frequency, and Gain metrics. The SHAP summary plot and variable importance ranking (Figure 4) emphasize the relevance of these features, showing their relative influence. Pneumonia and HF generally contributed toward higher predicted MV probabilities, whereas higher input volume, INR, and weight were associated with higher predicted probabilities of MV. Moreover, hematocrit demonstrated complex nonlinear relationships with the model output.

**Figure 5:**
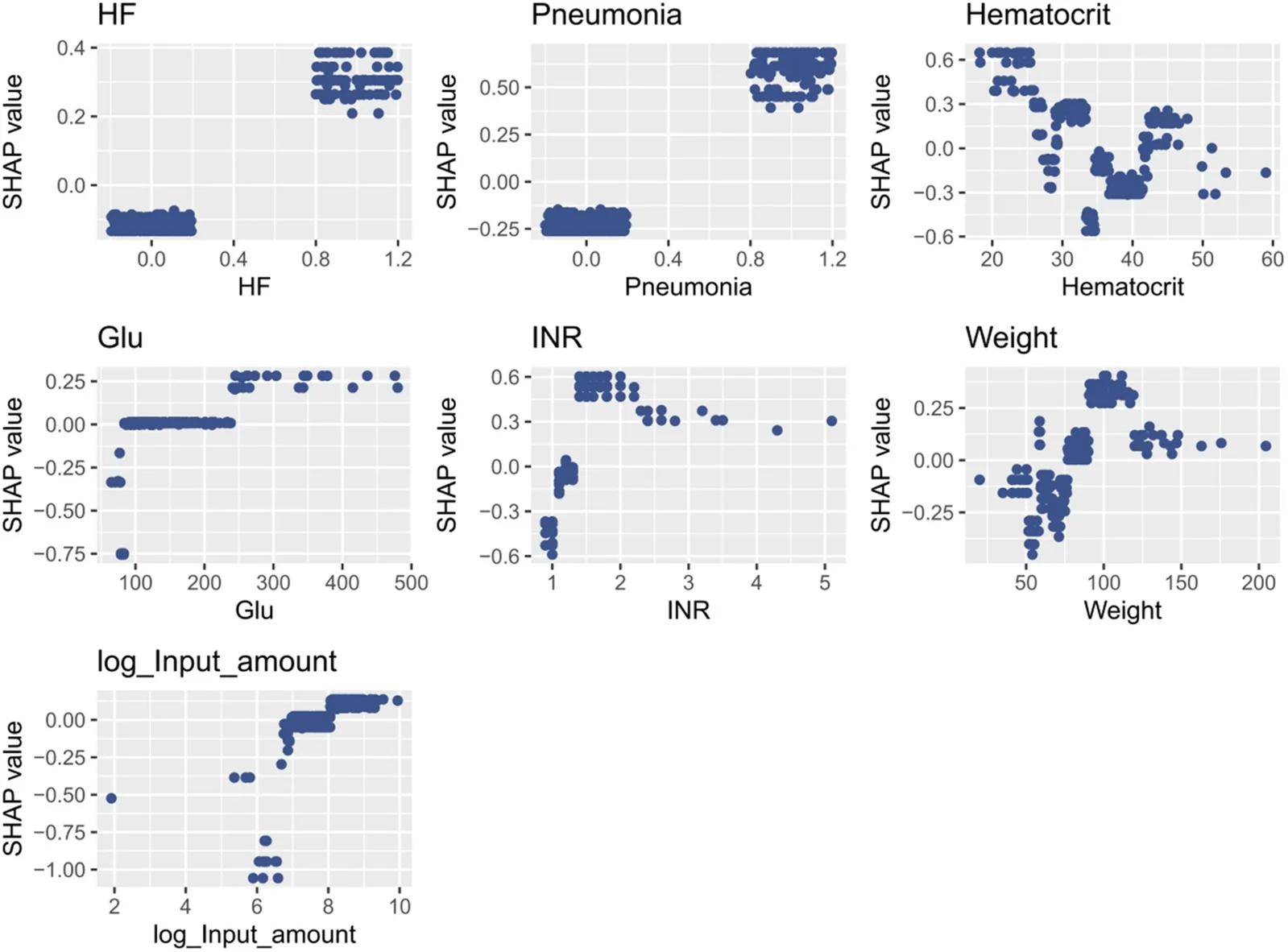
SHAP dependence plots for the seven predictor variables in the XGB model.

Figure 5 presents the SHAP dependence plots for seven predictor variables used in the XGB model. HF and pneumonia are high-importance variables associated with higher predicted probabilities of MV. Hematocrit showed a nonlinear, approximately U-shaped relationship with SHAP values; values around 35–45 generally had positive SHAP contributions, whereas values outside this range tended to have negative contributions. Higher INR, weight, and log input amount generally corresponded to more positive SHAP values, with apparent inflection points near 1.4, 75, and 8, respectively. It is worth noting that Glu had comparatively small mean absolute SHAP importance.

**Figure 6:**
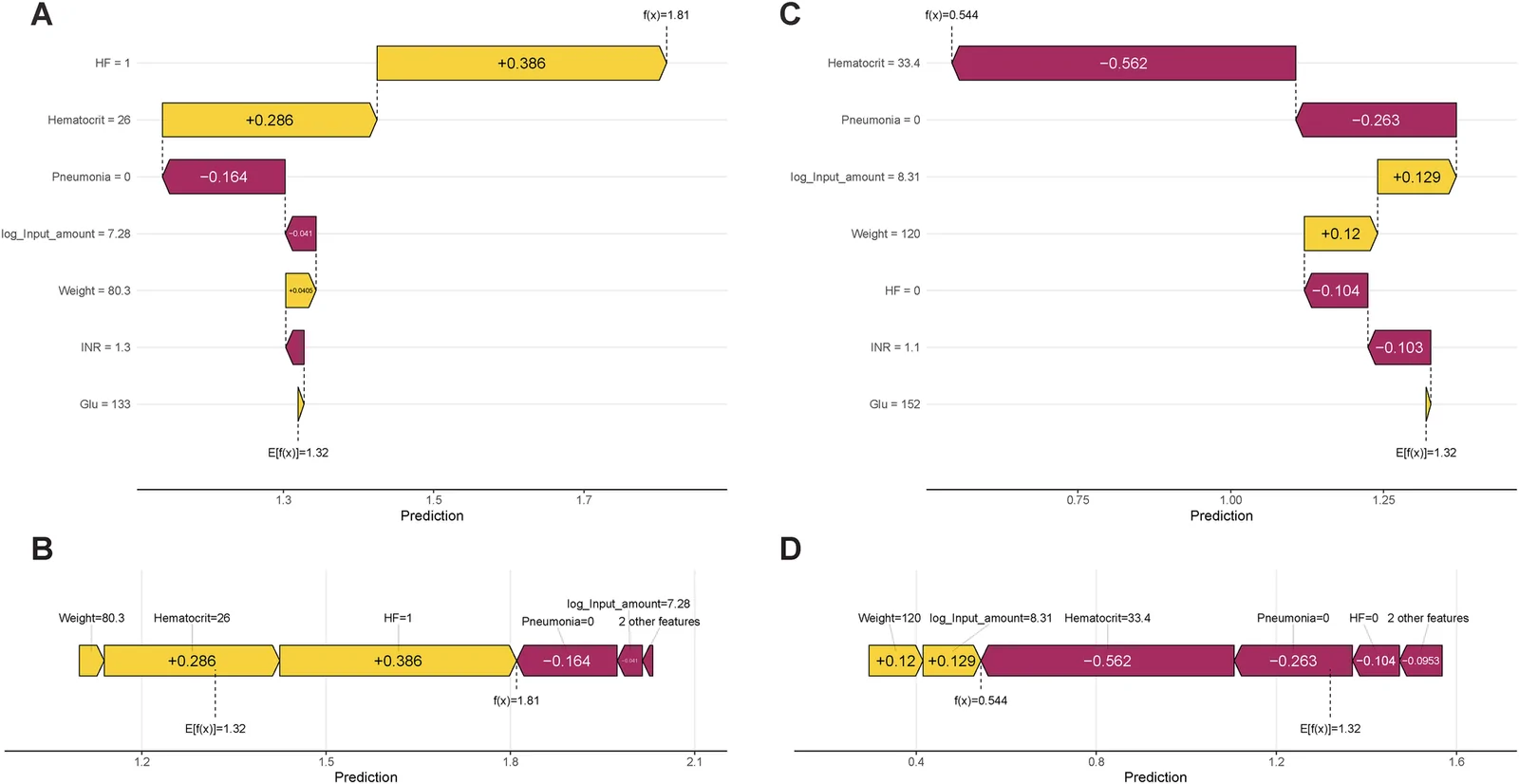
Individual predictions for two patients with embolic cerebral infarction. **(A)** Waterfall plot and **(B)** force plot for an MV case; **(C)** waterfall plot and **(D)** force plot for a non-MV case.

Waterfall plots in Figures 6A,B illustrate the prediction results for the need for MV support in patients with embolic cerebral infarction: when patients have HF and decreased hematocrit, the SHAP value rises to 1.81, indicating that HF combined with low hematocrit are the two largest positive contributors in this individual prediction. Although the absence of pneumonia contributed negatively to the model output (−0.164), it is insufficient to offset the positive model contributions from HF and low hematocrit. Figures 6C,D show the prediction results for the non-MV case: when hematocrit is within the normal range and there is no pneumonia, the model output (0.544) is lower than the baseline output (1.32), with an attribution direction consistent with the observed non-MV outcome.

The force plots shown in Figures 6B,D illustrate the SHAP values of different features that contribute to the final prediction outcomes. Yellow and purple bars represent positive and negative feature contributions, respectively, with longer bars indicating larger absolute contributions. These visualizations illustrate how individual model predictions were decomposed and do not establish causal effects.

## 4 Discussion

Traditionally, determining whether patients with embolic cerebral infarction need MV has mainly depended on general critical illness scores like the Glasgow Coma Scale (GCS) or clinical judgment, without specific quantitative predictive tools designed for this group (18, 19). This study created an interpretable predictive model that systematically uses machine learning combined with interpretability analysis to assess the necessity of MV in patients with embolic cerebral infarction. Four machine learning algorithms were employed to build the predictive models, with XGB showing the most favorable development performance but lower discrimination in validation. The preservation of discrimination, calibration, and clinical net benefit after excluding short-duration ventilation indicates that the primary findings were not driven by patients receiving only transient ventilatory support. The study also emphasized the significance of high-importance features such as pneumonia, hematocrit, and INR, which contributed materially to model predictions. To better understand the model, the SHAP method was used for visualization analysis, characterizing feature contributions to the fitted model. This approach helps clinicians grasp the reasoning behind the model's predictions, which is essential for fostering trust and supporting clinical decision-making. Likewise, another study assessed feature importance in machine learning models using SHAP values, demonstrating that this technique improves model interpretability and highlights the valuable role of SHAP in clinical applications, offering key insights for predictive modeling (20).

The 0.119 development-validation AUROC decrease, together with the nested-CV estimate and the 5–100 range of selected boosting rounds, indicates model-selection optimism and possible overfitting. Because nested resampling was conditional on the fixed preprocessed matrix, residual optimism from imputation, transformation, and predictor selection cannot be excluded. In this imbalanced cohort, raw accuracy was misleading: the all-MV benchmark achieved higher accuracy but zero specificity, whereas XGB improved balanced accuracy and probability accuracy; inverse-frequency weighting did not improve discrimination and worsened the Brier score.

For critically ill patients with embolic cerebral infarction, early assessment of MV requirement may support clinical decision-making and patient management. Timely risk stratification may facilitate life-support planning and resource allocation during a critical treatment window (21).

Pneumonia was the highest-ranked feature for predicting whether patients with embolic cerebral infarction require MV support. Severe cerebral infarction can lead to consciousness disturbances, swallowing difficulties, and weakened cough reflex, increasing the risk of aspiration and significantly raising the incidence of pneumonia (22, 23). Once pneumonia occurs, ventilation/perfusion mismatch and intrapulmonary shunting cause refractory hypoxemia, exacerbating cerebral edema and secondary brain injury. Pneumonia and brain injury form a “vicious cycle” (24). Therefore, pneumonia may identify patients with more severe respiratory and neurological compromise and may be associated with higher resource use, longer hospital stays, and mortality (25).

Pneumonia and cumulative input were recorded within the first 24 h, and exact pneumonia-onset and MV-initiation timestamps were unavailable. Their associations may therefore partly reflect evolving respiratory deterioration or clinical management rather than purely antecedent risk. Excluding both variables reduced discrimination, confirming their predictive contribution but not resolving chronology.

There is a nonlinear relationship between hematocrit and adverse outcomes, consistent with the findings of Roh, D. J. et al. (26). A high hematocrit indicates an increased proportion of red blood cells in the blood, leading to higher blood viscosity, which impairs cerebral blood circulation and may exacerbate cerebral edema and intracranial hypertension, affecting the brainstem respiratory center or causing secondary consciousness disorders, thereby indirectly increasing the risk of respiratory failure (27, 28). Increased blood viscosity raises peripheral vascular resistance and worsens cardiac afterload, potentially triggering or aggravating HF (29, 30). In this study, HF was associated with higher predicted MV probability, mainly because reduced cardiac output affects systemic oxygen delivery; under critical conditions with increased oxygen consumption, tissue hypoxia is more likely to occur, including respiratory muscle fatigue, thereby increasing the demand for MV (31, 32). Furthermore, in acute stroke patients, elevated hematocrit is often associated with imbalances in fluid intake and output. This study also found that higher cumulative fluid input was associated with higher predicted MV probability, which may lead to kidney dysfunction, electrolyte imbalances, and affect the metabolism and clearance of drugs (33, 34). These factors collectively weaken the patient's internal environment stability and organ functional reserve, making them more prone to decompensation when facing insults such as pneumonia and heart failure. Low hematocrit indicates reduced oxygen-carrying capacity of the body, impairing systemic oxygen delivery, exacerbating brain injury, promoting consciousness disorders, and causing respiratory muscle fatigue (35).

An abnormal INR reflects altered coagulation status. Such patients may have a heavier burden of cardiovascular and cerebrovascular diseases, and when embolic events occur, the infarct area may be larger or more prone to recurrence. In addition, abnormal INR has been associated with an increased risk of hemorrhagic transformation, which can sharply worsen cerebral edema, intracranial hypertension, and neurological damage, leading to rapid deterioration of consciousness and loss of airway protective reflexes (36, 37).

Weight is a meaningful predictor of the need for MV in patients with embolic stroke. Obesity is a clear risk factor for obstructive sleep apnea and hypoventilation syndrome (38). During the acute phase of ischemic stroke, due to impaired consciousness, use of sedatives, or a supine position, the risk of upper airway collapse increases sharply, making acute respiratory failure highly likely (39). In addition, patients admitted to the ICU are prone to respiratory muscle fatigue under conditions of high metabolism and high stress, which may increase the need for MV support (40, 41).

An interesting finding of this study is that although Glu has a limited contribution to the model's predictions, hyperglycemia is an important indicator of stress response and disease severity. In patients with embolic cerebral infarction, the occurrence of stress-induced hyperglycemia often reflects more severe central nervous system damage or a systemic inflammatory response. Overactivation of the sympathetic-adrenal axis leads to the release of catecholamines and cortisol, which not only raise Glu levels but also accelerate catabolism and suppress immune function, making patients more susceptible to secondary infections such as pneumonia (42, 43). This indicates that clinicians should be more vigilant about neurological deterioration and respiratory complications in patients with significantly elevated Glu levels (especially those without a history of diabetes) and should monitor and manage glucose as clinically appropriate.

## Limitations

This study also has limitations. First, the retrospective study design may lead to information bias, including data collection errors and missing data. Second, due to inherent limitations of the database, several clinical factors related to critically ill patients requiring MV, such as the use of sedatives, vasopressors, and proton pump inhibitors (PPIs), were not included in our predictive model. These unmeasured factors may have affected model performance. Future studies should conduct multicenter external validation and further verify factors that are crucial to the stability and performance of the model.

Third, the development-validation AUROC gap and wide variation in selected boosting rounds indicate possible overfitting and tuning instability. The nested-CV analysis was conditional on the fixed imputed and transformed matrix, so end-to-end optimism may remain. Fourth, exact pneumonia-onset and MV-initiation timestamps were unavailable; temporal overlap and information leakage cannot be excluded. Fifth, the cohort was identified with ICD-9-CM code 434.11 without a documented ICD-10-CM mapping, which may have reduced case capture after the coding transition. External temporal and multicenter validation using time-stamped predictors and outcomes is required before clinical implementation.

## 5 Conclusion

We developed four machine learning models (LR, DT, LGBM, XGB) and conducted a performance comparison. XGB had the most favorable overall validation profile among the evaluated models, although its lower validation than development AUROC indicates optimism. Analysis using the SHAP method revealed that indicators such as pneumonia, hematocrit, INR, and weight are closely associated with the need for MV support in patients with embolic cerebral infarction. These features may support first-day risk stratification, but the model should not be used as a stand-alone decision tool until temporal validity, calibration, and transportability are confirmed externally.

## Data Availability

The original contributions presented in the study are included in the article/Supplementary Material, further inquiries can be directed to the corresponding author.
