# Supplementary material for: Machine learning prediction models for mechanical ventilation requirement in patients with embolic stroke: a retrospective cohort study based on MIMIC-IV
Source: Front Med (Lausanne). 2026 Sep 9;13:1945368. doi: 10.3389/fmed.2026.1945368 (PMC13597239; doi:10.3389/fmed.2026.1945368)
Supplement: Supplementary file 1 [file Datasheet1.zip › Supplement files/Supplement figure.docx]

Supplement Figure 1 missing values for each variable.

Supplement Figure 2 Multicollinearity diagnostics performed before construction of the final model. (A): Spearman correlation matrix of the candidate predictors; (B):VIF assessment before final-model fitting

Note: Analyses used the development cohort (n=397). PT and INR were strongly correlated (Spearman rho=0.955) and had VIFs >20. PT was removed before final-model fitting; all remaining VIFs were ≤ 1.11. Dashed and dotted reference lines denote VIF thresholds of 5 and 10.

Supplement Figure 3 Refit of the final multivariable logistic regression model after exclusion of PT. (A):a forest plot of the refitted final model; (B): Comparison chart of standardized coefficients of various clinical indicators before and after PT exclusion.

Note: continuous predictors were scaled per one development-set SD and binary predictors were coded 1 vs 0. The final model was fitted in n=397 patients (307 events); validation AUROC=0.711 and Brier score=0.163.

Supplement Figure 4 Ventilation-duration heterogeneity and sensitivity-cohort construction. (A): Distribution among ventilated patients; (B): Cohort definition while preserving the original train-validation split; (C): Clinical heterogeneity across ventilation-duration subgroups (cell values in original units.

Note: All non-ventilated patients were retained. Sensitivity cohorts removed only ventilated patients below the specified duration threshold. MV: mechanical ventilation.

Supplement Figure 5 Validation performance after excluding short-duration mechanical ventilation. (A): ROC curves; (B): Calibration curves; (C):Decision-curve analysis.

Note: The original-cohort curve uses the supplied primary-model probabilities. Sensitivity models were re-fitted in the reduced training sets using the same seven predictors and reported XGB hyperparameters; validation split membership was preserved.

Supplement Figure 6 (A)Ranking of feature importance based on the Cover index; (B)Ranking of feature importance based on the Frequency index; (A)Ranking of feature importance based on the Gain index.
