# Supplementary material for: Machine learning prediction models for mechanical ventilation requirement in patients with embolic stroke: a retrospective cohort study based on MIMIC-IV
Source: Front Med (Lausanne). 2026 Sep 9;13:1945368. doi: 10.3389/fmed.2026.1945368 (PMC13597239; doi:10.3389/fmed.2026.1945368)
Supplement: Supplementary file 1 [file Datasheet1.zip › Supplement files/Supplement table.docx]

Supplement table 1 Comparison of general characteristics in the train group and test group.

| Variables | Total (n = 568) | test (n = 171) | train (n = 397) | Statistic | *P* |
| --- | --- | --- | --- | --- | --- |
|  |  |  |  |  |  |
| Hb, Mean ± SD | 11.68 ± 2.32 | 11.63 ± 2.15 | 11.71 ± 2.39 | t=-0.38 | 0.701 |
| Hematocrit, Mean ± SD | 34.94 ± 6.67 | 34.64 ± 6.30 | 35.07 ± 6.82 | t=-0.71 | 0.479 |
| SBP, M (Q₁, Q₃) | 134.00 (117.00, 154.25) | 134.00 (115.00, 151.50) | 134.00 (120.00, 155.00) | Z=-0.65 | 0.513 |
| WBC, M (Q₁, Q₃) | 10.50 (8.10, 13.50) | 10.60 (8.20, 13.90) | 10.50 (8.10, 13.10) | Z=-0.54 | 0.592 |
| RBC, M (Q₁, Q₃) | 3.94 (3.36, 4.39) | 3.84 (3.32, 4.31) | 3.97 (3.38, 4.43) | Z=-1.50 | 0.134 |
| PLT, M (Q₁, Q₃) | 211.00 (158.00, 275.25) | 211.00 (157.00, 274.00) | 211.00 (158.00, 276.00) | Z=-0.22 | 0.827 |
| RDW, M (Q₁, Q₃) | 14.10 (13.40, 15.00) | 14.10 (13.50, 14.90) | 14.10 (13.30, 15.20) | Z=-0.07 | 0.945 |
| Na, M (Q₁, Q₃) | 139.00 (137.00, 141.25) | 139.00 (137.00, 142.00) | 139.00 (137.00, 141.00) | Z=-0.13 | 0.897 |
| K, M (Q₁, Q₃) | 4.00 (3.70, 4.40) | 4.10 (3.70, 4.50) | 4.00 (3.60, 4.30) | Z=-2.58 | **0.010** |
| Ca, M (Q₁, Q₃) | 8.50 (8.00, 8.90) | 8.40 (8.00, 8.95) | 8.50 (8.00, 8.90) | Z=-0.58 | 0.561 |
| Cl, M (Q₁, Q₃) | 105.00 (102.00, 109.00) | 106.00 (102.00, 109.00) | 105.00 (102.00, 109.00) | Z=-0.47 | 0.638 |
| Glu, M (Q₁, Q₃) | 122.00 (105.00, 152.00) | 123.00 (106.50, 151.50) | 122.00 (104.00, 152.00) | Z=-0.38 | 0.702 |
| AG, M (Q₁, Q₃) | 14.00 (12.00, 16.00) | 14.00 (12.00, 16.00) | 14.00 (12.00, 16.00) | Z=-0.97 | 0.331 |
| PT, M (Q₁, Q₃) | 13.70 (12.47, 15.40) | 13.70 (12.50, 15.25) | 13.70 (12.40, 15.40) | Z=-0.29 | 0.772 |
| APTT, M (Q₁, Q₃) | 29.60 (26.48, 34.92) | 29.60 (26.20, 35.60) | 29.60 (26.50, 34.50) | Z=-0.39 | 0.696 |
| INR, M (Q₁, Q₃) | 1.20 (1.10, 1.40) | 1.20 (1.10, 1.40) | 1.20 (1.10, 1.40) | Z=-0.08 | 0.939 |
| BUN, M (Q₁, Q₃) | 18.00 (13.00, 27.00) | 18.00 (14.00, 28.00) | 18.00 (13.00, 26.00) | Z=-0.95 | 0.344 |
| Cr, M (Q₁, Q₃) | 0.90 (0.70, 1.30) | 1.00 (0.70, 1.30) | 0.90 (0.70, 1.20) | Z=-0.88 | 0.379 |
| HR, M (Q₁, Q₃) | 84.00 (72.00, 100.00) | 83.00 (70.50, 99.00) | 85.00 (73.00, 100.00) | Z=-1.01 | 0.312 |
| SBP, M (Q₁, Q₃) | 134.00 (117.00, 154.25) | 134.00 (115.00, 151.50) | 134.00 (120.00, 155.00) | Z=-0.65 | 0.513 |
| DBP, M (Q₁, Q₃) | 71.00 (59.00, 86.00) | 72.00 (57.50, 84.50) | 71.00 (60.00, 86.00) | Z=-0.60 | 0.549 |
| MAP, M (Q₁, Q₃) | 87.00 (74.00, 100.00) | 88.00 (72.50, 100.00) | 87.00 (75.00, 100.00) | Z=-0.69 | 0.493 |
| RR, M (Q₁, Q₃) | 18.00 (15.00, 22.00) | 18.00 (14.50, 22.00) | 18.00 (15.00, 22.00) | Z=-0.54 | 0.589 |
| SpO2, M (Q₁, Q₃) | 98.00 (96.00, 100.00) | 98.00 (96.00, 100.00) | 98.00 (96.00, 100.00) | Z=-0.92 | 0.360 |
| T, M (Q₁, Q₃) | 36.78 (36.44, 37.11) | 36.72 (36.39, 37.11) | 36.78 (36.44, 37.17) | Z=-1.72 | 0.085 |
| Weight, M (Q₁, Q₃) | 77.35 (64.55, 93.03) | 76.95 (64.35, 92.55) | 77.35 (64.95, 93.10) | Z=-0.51 | 0.613 |
| Age, M (Q₁, Q₃) | 71.50 (60.00, 83.00) | 72.00 (61.00, 82.50) | 71.00 (58.00, 83.00) | Z=-0.76 | 0.450 |
| Log Input Amount, M (Q₁, Q₃) | 7.95 (7.55, 8.39) | 7.98 (7.64, 8.42) | 7.92 (7.52, 8.35) | Z=-1.57 | 0.115 |
| Mechanical ventilation, n(%) |  |  |  | χ²=0.22 | 0.642 |
| No | 130 (22.89) | 37 (21.64) | 93 (23.43) |  |  |
| Yes | 438 (77.11) | 134 (78.36) | 304 (76.57) |  |  |
| Aspirin, n(%) |  |  |  | χ²=2.50 | 0.114 |
| No | 134 (23.59) | 33 (19.30) | 101 (25.44) |  |  |
| Yes | 434 (76.41) | 138 (80.70) | 296 (74.56) |  |  |
| Clopidogrel, n(%) |  |  |  | χ²=0.00 | 1.000 |
| No | 559 (98.42) | 168 (98.25) | 391 (98.49) |  |  |
| Yes | 9 (1.58) | 3 (1.75) | 6 (1.51) |  |  |
| Statin, n(%) |  |  |  | χ²=1.65 | 0.199 |
| No | 460 (80.99) | 144 (84.21) | 316 (79.60) |  |  |
| Yes | 108 (19.01) | 27 (15.79) | 81 (20.40) |  |  |
| Gender, n(%) |  |  |  | χ²=1.09 | 0.297 |
| Female | 280 (49.30) | 90 (52.63) | 190 (47.86) |  |  |
| Male | 288 (50.70) | 81 (47.37) | 207 (52.14) |  |  |
| Hypertension, n(%) |  |  |  | χ²=2.57 | 0.109 |
| No | 237 (41.73) | 80 (46.78) | 157 (39.55) |  |  |
| Yes | 331 (58.27) | 91 (53.22) | 240 (60.45) |  |  |
| Diabetes, n(%) |  |  |  | χ²=1.35 | 0.246 |
| No | 414 (72.89) | 119 (69.59) | 295 (74.31) |  |  |
| Yes | 154 (27.11) | 52 (30.41) | 102 (25.69) |  |  |
| HF, n(%) |  |  |  | χ²=1.95 | 0.162 |
| No | 424 (74.65) | 121 (70.76) | 303 (76.32) |  |  |
| Yes | 144 (25.35) | 50 (29.24) | 94 (23.68) |  |  |
| MI, n(%) |  |  |  | χ²=3.50 | 0.062 |
| No | 550 (96.83) | 162 (94.74) | 388 (97.73) |  |  |
| Yes | 18 (3.17) | 9 (5.26) | 9 (2.27) |  |  |
| Pneumonia, n(%) |  |  |  | χ²=6.19 | **0.013** |
| No | 397 (69.89) | 132 (77.19) | 265 (66.75) |  |  |
| Yes | 171 (30.11) | 39 (22.81) | 132 (33.25) |  |  |
| Hyperlipidemia, n(%) |  |  |  | χ²=2.01 | 0.156 |
| No | 331 (58.27) | 92 (53.80) | 239 (60.20) |  |  |
| Yes | 237 (41.73) | 79 (46.20) | 158 (39.80) |  |  |
| COPD, n(%) |  |  |  | χ²=0.11 | 0.745 |
| No | 561 (98.77) | 168 (98.25) | 393 (98.99) |  |  |
| Yes | 7 (1.23) | 3 (1.75) | 4 (1.01) |  |  |
| Split Value, n(%) |  |  |  | χ²=568.00 | **<.001** |
| test | 171 (30.11) | 171 (100.00) | 0 (0.00) |  |  |
| train | 397 (69.89) | 0 (0.00) | 397 (100.00) |  |  |
| t: t-test, Z: Mann-Whitney test, χ²: Chi-square test | | | | | |
| SD: standard deviation, M: Median, Q₁: 1st Quartile, Q₃: 3st Quartile | | | | | |

Supplementary Table 2 Variance inflation factors (VIFs) for independent variables in the multiple linear regression analysis of Mechanical Ventilation

| Variables | VIF before PT removal | Tolerance before PT removal | VIF after PT removal | Tolerance after PT removal |
| --- | --- | --- | --- | --- |
| HF | 1.082 | 0.924 | 1.064 | 0.940 |
| Pneumonia | 1.021 | 0.980 | 1.022 | 0.978 |
| Hematocrit | 1.100 | 0.909 | 1.111 | 0.900 |
| Glu | 1.021 | 0.980 | 1.017 | 0.983 |
| Weight | 1.051 | 0.952 | 1.048 | 0.954 |
| log_Input_amount | 1.071 | 0.934 | 1.061 | 0.942 |
| INR | 21.599 | 0.046 | 1.082 | 0.924 |
| PT | 21.473 | 0.047 | - | - |

Supplementary Table 3 Comparison of Brier score and PR-AUC indicators of each model on the training group and validation group

| Model | training group | validation group |
| --- | --- | --- |
|  | Brier score | Brier score |
| XGB | 0.1354 (0.1178-0.1555) | 0.1607 (0.1313-0.1926) |
| LR | 0.1540 (0.1363-0.1743) | 0.1630 (0.1319-0.1981) |
| LGBM | 0.1588 (0.1374-0.1838) | 0.1711 (0.1391-0.2086) |
| DT | 0.1298 (0.1095-0.1553) | 0.1873 (0.1517-0.2306) |

Supplementary Table 4 The Delong test results of each model

|  |  | DT | LM | XGB | LGBM |
| --- | --- | --- | --- | --- | --- |
| training group | DT | 1 | 0.349 | 0.021 | 0.186 |
|  | LM | 0.349 | 1 | 0.007 | 0.662 |
|  | XGB | 0.021 | 0.007 | 1 | 0.002 |
|  | LGBM | 0.186 | 0.662 | 0.002 | 1 |
| validation group | DT | 1 | 0.486 | 0.157 | 0.834 |
|  | LM | 0.486 | 1 | 0.892 | 0.622 |
|  | XGB | 0.157 | 0.892 | 1 | 0.521 |
|  | LGBM | 0.834 | 0.622 | 0.521 | 1 |

Supplementary Table 5 cohort characteristics and complete validation performance

| Analysis cohort | Total N | Validation N | Validation MV n | AUROC (95% CI) | AUPRC | Brier |
| --- | --- | --- | --- | --- | --- | --- |
| Primary cohort | 568 | 171 | 131 | 0.720 (0.637–0.801) | 0.897 | 0.161 |
| Exclude MV <12 h | 485 | 152 | 112 | 0.721 (0.631–0.800) | 0.882 | 0.174 |
| Exclude MV <24 h | 408 | 125 | 85 | 0.798 (0.709–0.869) | 0.877 | 0.168 |
