# Supplementary material for: Machine learning prediction models for mechanical ventilation requirement in patients with embolic stroke: a retrospective cohort study based on MIMIC-IV
Source: Front Med (Lausanne). 2026 Sep 9;13:1945368. doi: 10.3389/fmed.2026.1945368 (PMC13597239; doi:10.3389/fmed.2026.1945368)
Supplement: Supplementary file 2 [file Datasheet2.zip › supplement files/Figure_Sup.docx]

Supplementary Figure 1. Missingness of each candidate variable.

Supplementary Figure 2. First-24-hour predictor window and temporal-audit limitation. ICU admission was t0, and candidate predictors were summarized during [t0, t0 + 24 h). The supplied final analytic file contained a binary invasive-MV indicator for the ICU stay but did not retain exact MV-initiation or pneumonia-onset timestamps; therefore, strict post-landmark ordering cannot be verified. The temporal-sensitivity model excluded pneumonia and log input amount.

Note: The figure distinguishes the intended first-day risk-stratification use from a fully time-anchored prospective landmark model and avoids implying chronology that cannot be audited from the supplied patient-level file.

Supplementary Figure 3. Multicollinearity diagnostics before final-model construction. (A) Spearman correlation matrix of the candidate predictors; (B) VIF assessment before final-model fitting.

*Note: Analyses used the development cohort (n = 397). PT and INR were strongly correlated (Spearman rho = 0.955) and had VIFs >20. PT was removed before final-model fitting; all remaining VIFs were ≤ 1.11. Dashed and dotted reference lines denote VIF thresholds of 5 and 10.*

Supplementary Figure 4. Refit of the final multivariable logistic regression model after exclusion of PT. (A) Forest plot of the refitted final model; (B) comparison of standardized coefficients before and after PT exclusion.

*Note: continuous predictors were scaled per one development-set SD and binary predictors were coded 1 vs 0. The final model was fitted in n = 397 patients (307 events); validation AUROC = 0.711 and Brier score = 0.163.*

Supplementary Figure 5. Post-hoc repeated nested cross-validation analyses. (A) ROC curves based on averaged out-of-fold probabilities; (B) calibration curves; (C) boosting rounds selected for the full model by inner cross-validation across 50 outer folds; and (D) outer-fold AUROC distributions. The full model used seven predictors; the time-sensitive-predictor-exclusion model omitted pneumonia and log input amount; and the weighted model used inverse-frequency weights within each outer training fold. Nested resampling evaluated XGB fitting and boosting-round selection conditional on the supplied fixed preprocessed predictor matrix; imputation, transformation, and predictor selection were not repeated within folds.

Supplementary Figure 6. Ventilation-duration heterogeneity and sensitivity-cohort construction. (A) Distribution among ventilated patients; (B) cohort definition while preserving the original development-validation split; and (C) clinical characteristics across ventilation-duration subgroups (cell values in original units).

*Note: All non-ventilated patients were retained. Sensitivity cohorts removed only ventilated patients below the specified duration threshold. MV: mechanical ventilation.*

Supplementary Figure 7. Validation performance after excluding short-duration mechanical ventilation. (A) ROC curves; (B) calibration curves; and (C) decision-curve analysis.

*Note: The original-cohort curve uses the supplied primary-model probabilities. Sensitivity models were re-fitted in the reduced development sets using the same seven predictors and reported XGB hyperparameters; validation split membership was preserved.*

Supplementary Figure 8. (A) Ranking of feature importance based on the Cover index; (B) ranking based on the Frequency index; and (C) ranking based on the Gain index.
