# Supplementary material for: Machine learning prediction models for mechanical ventilation requirement in patients with embolic stroke: a retrospective cohort study based on MIMIC-IV
Source: Front Med (Lausanne). 2026 Sep 9;13:1945368. doi: 10.3389/fmed.2026.1945368 (PMC13597239; doi:10.3389/fmed.2026.1945368)
Supplement: Supplementary file 2 [file Datasheet2.zip › supplement files/Table_Sup.docx]

**Supplementary Table 1. Comparison of baseline characteristics between the development and validation cohorts.**

| **Variables** | **Total (n = 568)** | **Validation (n = 171)** | **Development (n = 397)** | **Statistic** | **P** |
| --- | --- | --- | --- | --- | --- |
| Hb, Mean ± SD | 11.68 ± 2.32 | 11.63 ± 2.15 | 11.71 ± 2.39 | t=-0.38 | 0.701 |
| Hematocrit, Mean ± SD | 34.94 ± 6.67 | 34.64 ± 6.30 | 35.07 ± 6.82 | t=-0.71 | 0.479 |
| SBP, M (Q₁, Q₃) | 134.00 (117.00, 154.25) | 134.00 (115.00, 151.50) | 134.00 (120.00, 155.00) | Z=-0.65 | 0.513 |
| WBC, M (Q₁, Q₃) | 10.50 (8.10, 13.50) | 10.60 (8.20, 13.90) | 10.50 (8.10, 13.10) | Z=-0.54 | 0.592 |
| RBC, M (Q₁, Q₃) | 3.94 (3.36, 4.39) | 3.84 (3.32, 4.31) | 3.97 (3.38, 4.43) | Z=-1.50 | 0.134 |
| PLT, M (Q₁, Q₃) | 211.00 (158.00, 275.25) | 211.00 (157.00, 274.00) | 211.00 (158.00, 276.00) | Z=-0.22 | 0.827 |
| RDW, M (Q₁, Q₃) | 14.10 (13.40, 15.00) | 14.10 (13.50, 14.90) | 14.10 (13.30, 15.20) | Z=-0.07 | 0.945 |
| Na, M (Q₁, Q₃) | 139.00 (137.00, 141.25) | 139.00 (137.00, 142.00) | 139.00 (137.00, 141.00) | Z=-0.13 | 0.897 |
| K, M (Q₁, Q₃) | 4.00 (3.70, 4.40) | 4.10 (3.70, 4.50) | 4.00 (3.60, 4.30) | Z=-2.58 | 0.010 |
| Ca, M (Q₁, Q₃) | 8.50 (8.00, 8.90) | 8.40 (8.00, 8.95) | 8.50 (8.00, 8.90) | Z=-0.58 | 0.561 |
| Cl, M (Q₁, Q₃) | 105.00 (102.00, 109.00) | 106.00 (102.00, 109.00) | 105.00 (102.00, 109.00) | Z=-0.47 | 0.638 |
| Glu, M (Q₁, Q₃) | 122.00 (105.00, 152.00) | 123.00 (106.50, 151.50) | 122.00 (104.00, 152.00) | Z=-0.38 | 0.702 |
| AG, M (Q₁, Q₃) | 14.00 (12.00, 16.00) | 14.00 (12.00, 16.00) | 14.00 (12.00, 16.00) | Z=-0.97 | 0.331 |
| PT, M (Q₁, Q₃) | 13.70 (12.47, 15.40) | 13.70 (12.50, 15.25) | 13.70 (12.40, 15.40) | Z=-0.29 | 0.772 |
| APTT, M (Q₁, Q₃) | 29.60 (26.48, 34.92) | 29.60 (26.20, 35.60) | 29.60 (26.50, 34.50) | Z=-0.39 | 0.696 |
| INR, M (Q₁, Q₃) | 1.20 (1.10, 1.40) | 1.20 (1.10, 1.40) | 1.20 (1.10, 1.40) | Z=-0.08 | 0.939 |
| BUN, M (Q₁, Q₃) | 18.00 (13.00, 27.00) | 18.00 (14.00, 28.00) | 18.00 (13.00, 26.00) | Z=-0.95 | 0.344 |
| Cr, M (Q₁, Q₃) | 0.90 (0.70, 1.30) | 1.00 (0.70, 1.30) | 0.90 (0.70, 1.20) | Z=-0.88 | 0.379 |
| HR, M (Q₁, Q₃) | 84.00 (72.00, 100.00) | 83.00 (70.50, 99.00) | 85.00 (73.00, 100.00) | Z=-1.01 | 0.312 |
| DBP, M (Q₁, Q₃) | 71.00 (59.00, 86.00) | 72.00 (57.50, 84.50) | 71.00 (60.00, 86.00) | Z=-0.60 | 0.549 |
| MAP, M (Q₁, Q₃) | 87.00 (74.00, 100.00) | 88.00 (72.50, 100.00) | 87.00 (75.00, 100.00) | Z=-0.69 | 0.493 |
| RR, M (Q₁, Q₃) | 18.00 (15.00, 22.00) | 18.00 (14.50, 22.00) | 18.00 (15.00, 22.00) | Z=-0.54 | 0.589 |
| SpO2, M (Q₁, Q₃) | 98.00 (96.00, 100.00) | 98.00 (96.00, 100.00) | 98.00 (96.00, 100.00) | Z=-0.92 | 0.360 |
| T, M (Q₁, Q₃) | 36.78 (36.44, 37.11) | 36.72 (36.39, 37.11) | 36.78 (36.44, 37.17) | Z=-1.72 | 0.085 |
| Weight, M (Q₁, Q₃) | 77.35 (64.55, 93.03) | 76.95 (64.35, 92.55) | 77.35 (64.95, 93.10) | Z=-0.51 | 0.613 |
| Age, M (Q₁, Q₃) | 71.50 (60.00, 83.00) | 72.00 (61.00, 82.50) | 71.00 (58.00, 83.00) | Z=-0.76 | 0.450 |
| Log Input Amount, M (Q₁, Q₃) | 7.95 (7.55, 8.39) | 7.98 (7.64, 8.42) | 7.92 (7.52, 8.35) | Z=-1.57 | 0.115 |
| Mechanical ventilation, n(%) |  |  |  | χ²=0.22 | 0.642 |
| No | 130 (22.89) | 40 (23.39) | 90 (22.67) |  |  |
| Yes | 438 (77.11) | 131 (76.61) | 307 (77.33) |  |  |
| Aspirin, n(%) |  |  |  | χ²=2.50 | 0.114 |
| No | 134 (23.59) | 33 (19.30) | 101 (25.44) |  |  |
| Yes | 434 (76.41) | 138 (80.70) | 296 (74.56) |  |  |
| Clopidogrel, n(%) |  |  |  | χ²=0.00 | 1.000 |
| No | 559 (98.42) | 168 (98.25) | 391 (98.49) |  |  |
| Yes | 9 (1.58) | 3 (1.75) | 6 (1.51) |  |  |
| Statin, n(%) |  |  |  | χ²=1.65 | 0.199 |
| No | 460 (80.99) | 144 (84.21) | 316 (79.60) |  |  |
| Yes | 108 (19.01) | 27 (15.79) | 81 (20.40) |  |  |
| Gender, n(%) |  |  |  | χ²=1.09 | 0.297 |
| Female | 280 (49.30) | 90 (52.63) | 190 (47.86) |  |  |
| Male | 288 (50.70) | 81 (47.37) | 207 (52.14) |  |  |
| Hypertension, n(%) |  |  |  | χ²=2.57 | 0.109 |
| No | 237 (41.73) | 80 (46.78) | 157 (39.55) |  |  |
| Yes | 331 (58.27) | 91 (53.22) | 240 (60.45) |  |  |
| Diabetes, n(%) |  |  |  | χ²=1.35 | 0.246 |
| No | 414 (72.89) | 119 (69.59) | 295 (74.31) |  |  |
| Yes | 154 (27.11) | 52 (30.41) | 102 (25.69) |  |  |
| HF, n(%) |  |  |  | χ²=1.95 | 0.162 |
| No | 424 (74.65) | 121 (70.76) | 303 (76.32) |  |  |
| Yes | 144 (25.35) | 50 (29.24) | 94 (23.68) |  |  |
| MI, n(%) |  |  |  | χ²=3.50 | 0.062 |
| No | 550 (96.83) | 162 (94.74) | 388 (97.73) |  |  |
| Yes | 18 (3.17) | 9 (5.26) | 9 (2.27) |  |  |
| Pneumonia, n(%) |  |  |  | χ²=6.19 | 0.013 |
| No | 397 (69.89) | 132 (77.19) | 265 (66.75) |  |  |
| Yes | 171 (30.11) | 39 (22.81) | 132 (33.25) |  |  |
| Hyperlipidemia, n(%) |  |  |  | χ²=2.01 | 0.156 |
| No | 331 (58.27) | 92 (53.80) | 239 (60.20) |  |  |
| Yes | 237 (41.73) | 79 (46.20) | 158 (39.80) |  |  |
| COPD, n(%) |  |  |  | χ²=0.11 | 0.745 |
| No | 561 (98.77) | 168 (98.25) | 393 (98.99) |  |  |
| Yes | 7 (1.23) | 3 (1.75) | 4 (1.01) |  |  |
| t: t-test, Z: Mann-Whitney test, χ²: Chi-square test | | | | | |
| SD: standard deviation, M: median, Q₁: first quartile, Q₃: third quartile | | | | | |

**Supplementary Table 2. Variance inflation factors and tolerance values before and after PT removal.**

| **Variables** | **VIF before PT removal** | **Tolerance before PT removal** | **VIF after PT removal** | **Tolerance after PT removal** |
| --- | --- | --- | --- | --- |
| HF | 1.082 | 0.924 | 1.064 | 0.940 |
| Pneumonia | 1.021 | 0.980 | 1.022 | 0.978 |
| Hematocrit | 1.100 | 0.909 | 1.111 | 0.900 |
| Glu | 1.021 | 0.980 | 1.017 | 0.983 |
| Weight | 1.051 | 0.952 | 1.048 | 0.954 |
| Log input amount | 1.071 | 0.934 | 1.061 | 0.942 |
| INR | 21.599 | 0.046 | 1.082 | 0.924 |
| PT | 21.473 | 0.047 | - | - |

**Supplementary Table 3. Brier scores of the four models in the development and validation sets.**

| **Model** | **Development set** | **Validation set** |
| --- | --- | --- |
|  | **Brier score** | **Brier score** |
| XGB | 0.1354 (0.1178-0.1555) | 0.1607 (0.1313-0.1926) |
| LR | 0.1540 (0.1363-0.1743) | 0.1630 (0.1319-0.1981) |
| LGBM | 0.1588 (0.1374-0.1838) | 0.1711 (0.1391-0.2086) |
| DT | 0.1298 (0.1095-0.1553) | 0.1873 (0.1517-0.2306) |

**Supplementary Table 4. Pairwise DeLong test P values for the four models.**

|  |  | **DT** | **LR** | **XGB** | **LGBM** |
| --- | --- | --- | --- | --- | --- |
| development group | DT | 1 | 0.349 | 0.021 | 0.186 |
|  | LR | 0.349 | 1 | 0.007 | 0.662 |
|  | XGB | 0.021 | 0.007 | 1 | 0.002 |
|  | LGBM | 0.186 | 0.662 | 0.002 | 1 |
| validation group | DT | 1 | 0.486 | 0.157 | 0.834 |
|  | LR | 0.486 | 1 | 0.892 | 0.622 |
|  | XGB | 0.157 | 0.892 | 1 | 0.521 |
|  | LGBM | 0.834 | 0.622 | 0.521 | 1 |

**Supplementary Table 5. Repeated nested cross-validation robustness analyses conditional on the fixed preprocessed predictor matrix.**

| **Analysis** | **Predictors** | **AUROC (95% CI)** | **AUPRC (95% CI)** | **Brier (95% CI)** | **Calibration intercept (95% CI)** | **Calibration slope (95% CI)** | **Median boosting rounds (range)** |
| --- | --- | --- | --- | --- | --- | --- | --- |
| Full unweighted XGB | HF, pneumonia, hematocrit, glucose, weight, log input, INR | 0.713 (0.665-0.757) | 0.898 (0.875-0.918) | 0.161 (0.153-0.169) | -0.041 (-0.361 to 0.235) | 1.033 (0.796 to 1.322) | 20 (5-100) |
| Time-sensitive-predictor-exclusion XGB | HF, hematocrit, glucose, weight, INR | 0.654 (0.595-0.703) | 0.851 (0.817-0.881) | 0.168 (0.161-0.176) | 0.225 (-0.156 to 0.586) | 0.785 (0.485 to 1.108) | 20 (5-100) |
| Inverse-frequency weighted XGB | Same seven predictors as the full model | 0.717 (0.668-0.761) | 0.897 (0.873-0.918) | 0.200 (0.187-0.214) | 1.038 (0.965 to 1.107) | 0.955 (0.729 to 1.223) | 20 (5-100) |

Note: Repeated nested fivefold cross-validation used 10 repeats (50 outer test folds). Inner stratified fivefold resampling selected boosting rounds from 5, 10, 15, 20, 25, 30, 40, 50, 75, and 100. The outer random seed was 1234; the inner seed was 5678 plus the outer-fold index. Performance was calculated from the average of each patient’s 10 out-of-fold probabilities. The analysis was conditional on the supplied fixed preprocessed predictor matrix; imputation, transformation, and predictor selection were not re-estimated within folds. The paired AUROC difference between the full and time-sensitive-predictor-exclusion models was 0.059 (95% CI, 0.024-0.095; bootstrap P < 0.001). The difference between the full and weighted models was -0.004 (95% CI, -0.015 to 0.007; P = 0.434).

**Supplementary Table 6. Validation-set class-imbalance benchmark: XGB versus an all-MV classifier.**

| **Metric** | **XGB** | **All-MV benchmark** | **Interpretation** |
| --- | --- | --- | --- |
| Accuracy | 0.637 | 0.766 | Raw accuracy favors the majority-class rule |
| Sensitivity | 0.618 | 1.000 | All-MV identifies every MV case by definition |
| Specificity | 0.700 | 0.000 | All-MV cannot identify any non-MV case |
| Precision | 0.871 | 0.766 | Positive predictive value |
| F1 score | 0.723 | 0.868 | Inflated for the benchmark because MV prevalence is high |
| Balanced accuracy | 0.659 | 0.500 | Accounts equally for sensitivity and specificity |
| AUROC | 0.720 (0.637-0.801) | 0.500 | Discrimination across thresholds |
| AUPRC | 0.897 | 0.766 | Benchmark equals outcome prevalence |
| Brier score | 0.161 | 0.234 | Lower values indicate more accurate probabilities |

*Note: Validation n = 171, including 131 MV and 40 non-MV patients. XGB accuracy was reconstructed from the reported confusion-matrix metrics (81 true positives and 28 true negatives). The benchmark assigns probability 1 to every patient.*

**Supplementary Table 7. Cohort characteristics and validation performance in ventilation-duration sensitivity analyses.**

| **Analysis cohort** | **Total N** | **Validation N** | **Validation MV, n** | **AUROC (95% CI)** | **AUPRC** | **Brier** |
| --- | --- | --- | --- | --- | --- | --- |
| Primary cohort | 568 | 171 | 131 | 0.720 (0.637–0.801) | 0.897 | 0.161 |
| Exclude MV <12 h | 485 | 152 | 112 | 0.721 (0.631–0.800) | 0.882 | 0.174 |
| Exclude MV <24 h | 408 | 125 | 85 | 0.798 (0.709–0.869) | 0.877 | 0.168 |
